# Report on the Immediate or Exciting Cause of the Asiatic Cholera, Which Suddenly Appeared with Great Malignancy, on Ellicott Street, in Buffalo, on Monday, Tuesday, and Wednesday, the 26th, 27th, and 28th of July, 1852

**Published:** 1852-09

**Authors:** Frank H. Hamilton


ART. IV.—*Report on the immediate or exciting cause of the Asiatic Cholera, which suddenly appeared with great malignancy, on Ellicott street, in Buffalo, on Monday, Tuesday, and Wednesday, the* 26*th*, 27*th, and* 28*th of July*, 1852. *Read before the* "*Buffalo Medical Association*," *at its regular meeting, Aug.* 2*d*, 1852. By FRANK H. HAMILTON, M. D.

On Monday, Tuesday, and Wednesday of last week, the 26th, 27th, and 28th ult., the Asiatic Cholera made a sudden and fearful irruption into one of our most healthy neighborhoods: and after a brief but fatal sojourn its departure was announced almost as suddenly as its accession.

In this simple event, those who have read the history, or themselves witnessed the eccentric wanderings of this scourge, will see nothing new or extraordinary. The circumstances which herald its approach, and the laws which determine its direction, have hitherto been only feebly ascertained. It has defied everywhere, at certain times, quarantines, cordon sanitaires, climate, season, and meteoric influences — no person can be so vigilant and prudent in his habits as to secure an insurance — no place is so favored as to possess an unqualified immunity. If to-day it seems to be restrained by natural or artificial limits, to-morrow it may overleap all barriers, and swift as a passing cloud, it may throw its shadows successively upon the valley and the mountains, upon the city and the plain.

Yet, notwithstanding all its apparent irregularities, no intelligent man doubts, I presume, that the Asiatic cholera possesses habitudes as precise and distinctive as those which belong to any other disease; and that all its movements are directed by laws as exact and inviolable as those which control any other events. All its paradoxes are the common results of consistent causes. It never makes a foray without provocation, or retires until compelled by overpowering forces. The causes exist, and it only remains for us to ascertain, and having ascertained, to remove them, that the effects may cease.

I remind you, gentlemen, of these indisputable truths, which no one is simple enough to question, because we do not always act as if we fully believed them. We see in the cholera so many phenomena yet unexplained, that we are inclined to distrust every thing pertaining to it — and to doubt those rational explanations which fortunate coincidences, and careful investigations have furnished us. We become perplexed and bewildered in its seeming inconsistencies, and finally declare the whole a mystery — and refuse any longer to accept of arguments. In this way I seek to explain the incredulity which some of my professional brethren, whose opinions I am accustomed to respect, have manifested in relation to the immediate source of the cholera as it appeared on the 26th, 27th, and 28th ult., in Ellicott street.

To me it seems too palpable to admit of a question, that it had its cause in the ditch which, commenced at Eagle street on Saturday, was finished through to South Division on Wednesday morning.

Permit me, gentlemen, if you please, to give my reasons for this belief.

Not many years ago a marsh occupied the ground where this street is built, covered with a deep, soft, alluvial mould. The marsh extended from near Washington street to about where now Michigan street lies, and from Goodell street to Swan. It had its outlet toward the corner of Swan and Michigan, or in that direction. This marsh was the result of a peculiar formation of the clay bed, which to the depth of ten or twenty feet underlies nearly all that portion of the city which is east of Main street and below High street. The strata having a dip from Main and Michigan, from Goodell and Swan toward a common center. To this clay basin, only partly filled in its deepest portions with sand, there was no actual outlet, except the slight depression toward the south-east: and it remained therefor, until intersected by ditches, the depot for all the surface drainage of the higher neighborhoods — a general recepticle for water, alluvium and sewers. Where the Irish catholic church now stands the water formed a pond, and lower down along where Ellicott extends toward South Division the ground was nearly, but not quite as low.

Upon this soil much of that portion of the town is built; for in paving the streets, with few exceptions, none of the surface earth was removed, but the sand was deposited for pavements, above it. The streets thus became higher than the adjoining lots, and the water being thrown back upon them the owners found it necessary to fill them up. So this whole bed of alluvium, &c., was at length buried up, and there it has remained to the present time. No less rich and fertile, and redolent of disease, however, to-day, than before it was inhumed — when it was regarded as unsafe for any family to live within the reach of its miasms.

Since the pavements were laid, the lots filled up and the sewers made, this part of the town has been as healthy as any of those portions which are underlaid with clay — indeed much more so, I think. Ellicott street, especially, and particularly at this southern extremity, has been regarded as healthy. In 1849, fewer deaths from cholera occurred in this street than in Washington, Elm, Oak or in any other parallel street of equal length; and I am informed by a resident that not one death occurred from this cause, in that portion of the street of which we are now speaking. And this fact may be explained by the size and comfort of the dwellings, which are mostly brick; by the neatness and spaciousness of their yards, which affords them sufficient ventilation; by the cleanliness of their street, and the completeness of their sewerage, which last possesses also, I am told, this remarkable advantage over other sewers, that it has running through it most of the year, if not constantly, a fresh current of water, which finds its supply in springs around the foot of court house hill, and from other parts of the clay basin in that vicinity.

In short, for several years the occupants of these houses have enjoyed that immunity from epidemic and other diseases which the science of etiology would have taught us to expect for them, and to which their own diligence in the abatement of the usual causes has eminently entitled them.

During the present season the reputation of this locality for healthfulness remained unchanged. While the cholera has been here and there in other portions of the city, and especially in the low parts of the town, and at the eastern extremity toward the "Hydraulics," where the same geological structure prevails, viz., a clay subsoil, with less pavements and sewerage, and less comfort in the style of the tenements, still here were no indications of disease; not even the ordinary diseases of summer were known to have prevailed.

The long and persevering drought, with an unusual degree of heat brought no change.

On Saturday, July 24th, a ditch was commenced at Eagle street, four and a half deep, and two feet wide, for the purpose of laying water pipes. The work was regularly carried on through Saturday, Monday, Tuesday, and a part of Wednesday forenoon. On monday night it was partly open near the corner of North Division and Ellicott. Wednesday morning it was opened to South Division street.

The length of the ditch was about 200 yards, and the number of dwellings fronting upon the street, from Eagle to South Division was twenty.

The soil through which the ditch was dug, was, directly underneath the pavement, a coarse sand of about one foot in depth, then a rich loam averaging about one foot also; and underneath this, sand of a reddish or yellow color, either coarse or fine at different points. The clay bed beneath was not reached.

Mr. I. P. occupies a recently built and very comfortably constructed brick house, on the north-west corner of Ellicott and North Division streets. His house is well ventilated and his cellars sufficiently drained.

On Monday evening, July 26th, Mrs. P., who had been in feeble health for some months, but with no intestinal disease, was attacked with a slight diarrhœa. On Tuesday it returned with increased severity, and on Wednesday during the forenoon, cholera was distinctly announced. She died Friday morning at 2 o'clock.

No other members of the family were attacked.

Mr. O. H. P. W., with his family, consisting of himself and wife, three children, an apprentice and a servant girl, lived in the new brick house, No. 35 Ellicott, on the east side, and three doors north of North Division street. The house is two stories and a half high, with an open, clean yard, and is well constructed for ventilation. The cellar is well drained.

Monday night the family all slept in the house, with more or less of their windows open.

Tuesday morning Mr. W. arose feeling ill, and with a diarrhœa. During the day he had ten or twelve copious watery evacuations, but having taken medicine and rest, the disease was arrested, and never returned with severity.

On the same morning, Tuesday, Mrs. W. awoke with a severe headache, and early in the afternoon a diarr œa commenced, which followed her, accompanied with unequivocal symptoms of Asiatic cholera, until the next day, at 5, P. M., when she died. The signs of cholera did not supervene, however, until about midnight of Tuesday.

John Berry, the apprentice, slept at Mr. W.'s Monday night, and Tuesday morning went to the Falls — returning Tuesday night on the boat, he felt sea-sick, as he thought. He reached home between 9 and 10, and retired without mentioning his illness. In the night, about 11 o'clock, he had occasion to get up a d the family discovered his condition. From this time until 10, A. M., of Wednesday, when he died, he had unequivocal Asiatic cholera.

No other members of the family fell sick on Tuesday.

It has been thought that the currant pudding eaten by some of the family on Tuesday, at dinner, might have caused the cholera; but Berry was not at dinner at Mr. W.'s. Mr. W.'s diarrhœa commenced in the morning; and Mrs. W. awoke quite ill, although her diarrhœa did not commence until after dinner. Nor was it a thing unusual for the family to have currant puddings and other similar fruit deserts at dinner.

Wednesday morning I. W., about two years old, was attacked as early as 6 o'clock, with vomiting and purging. In the afternoon he was sent out of the house, and died on the Sunday or Monday following, the 1st or 2d of August. His symptoms were finally those of dysentery.

H. W., aged about four or five years, was atta ked at his father's house, as early as 12 o'clock, at noon, of Wednesday — perhaps earlier, and died about 5 o'clock the next morning.

M. W. was attacked some time during the day on Wednesday — probably in the forenoon. He was removed from the house, and is now, August 2d, convalescing.

Mr. E. C., aged about sixty years, was attacked at 2 o'clock, A. M., of Wednesday, and died at half past 1 of the same day. He occupied the house No. 27, east side of Ellicott.

Mrs. C., his wife, was attacked about 11, A. M., of same day, Wednesday, and died at 11, A. M., on Thursday.

Miss C., daughter of Mr. C., aged twenty-four years, returned from a visit of a week at the Falls, on Saturday afternoon. Tuesday, before the cholera had appeared in the family, she felt quite ill, but had no cholera symptoms. Thursday she left town with the family for Lima, and was taken with Asiatic cholera at this latter place, on Friday evening, and died about one week after.

Elizabeth Tres, the servant girl of Mr. W., (35 Ellicott,) aged seventeen years, was attacked Wednesday night, or early Thursday morning, and died at about 5 o'clock, P. M., on Thursday.

Mr. J. R. E. resides at 31 Ellicott, N. E. corner of North Division and Ellicott, in a two and a half story frame house. Mr. E. and two of his children were attacked, I am informed, with diarrhœa and dysenteric symptoms, on Wednesday or Thursday, and are recovering.

Mr. E. T. B., resides at 24 Ellicott, on the west side. His daughter, aged nine years, was attacked with diarrhœa, copious and watery, on Thursday, P. M., at about five or six o'clock.

Mrs. B. was attacked Thursday at midnight, in the same manner. Both are now convalescent.

Mr. M. resides at 25 Ellicott, east side. Mrs. M. was attacked Thursday morning at three o'clock, with nausea, prostration and diarrhœa.

Her daughter had a slight illness of the same character and at the same time!

It is entirely pertinent to include in this enumeration the case of Mrs. T., who having eaten freely of cherries on Sunday, was attacked at her house, No. 22 Ellicott, west side, with a very profuse diarrhœa of the rice water character; and the same returned, with more or less severity, on two or three successive days. I think on Monday, Tuesday, and Wednesday.

Mr. P. resides at 37 Ellicott, and Mrs. P. was ill on Sunday with diarrhœa; and the servant girl fell sick about Thursday noon, with the same malady.

The dwellings to which I have referred, are all, with one exception, brick, and generally new. They are also, I believe, all well drained and well ventilated, having generally spacious yards, and in most cases being detached from the adjacent buildings.

I have heard of one other case as having occurred a few doors above Mr. W.'s, on Wednesday, but I have not been able to learn the facts.

We have thus occurring within the distance of a few rods each way from the centre of the ditch, near the intersection of North Division with Ellicott, nineteen cases of diarrhœa, with manifest cholera tendency, (all being so ill as to require medical attendance) or with actual cholera; and of these, nine have died. Of the six whose illness commenced on or before Tuesday, four have died. Of the six attacked on Wednesday, five have died; and of seven attacked on Thursday, none have died. Since Thursday no new cases have occurred in that neighborhood, In twenty families living upon the street the epidemic has shown itself in nine or ten, possibly in others.

Early Thursday morning the attention of the Board of Health was called to the probable connection between the opening of the ditch and the existence o the cholera in this street, and they promptly issued an order to have it closed. The contractor had, however, commenced replacing the earth at the request of several residents of the street; and by one o'clock, P. M., of Thursday, July 29th, it was completely closed.

My conviction is, under all these circumstance, that these cases, all had their source, more or less directly in the miasms from the ditch. I have no doubt other causes may have concurred and materially promoted the result—eating sour, or unripe fruit — alarm — even contagion I admit; yet neither one nor all of these are sufficient to explain many of the cases. They did not all eat fruit — several were attacked simultaneously in the night before it was known that the cholera was in the neighborhood — children and almost infants were in many instances its subjects.

The weather was very warm, and immediately when this old bed was opened and brought to the surface, a rapid decomposition and elimination commenced. During the day the heat of the sun so rarified the air, that the mephitic or poisonous gases arose rapidly and were borne off; but during the night, when most of the attacks commenced, these exhalations settled and hung upon the houses and their unsuspecting occupants, like the heavy vapors of a pit. The men who dug the ditch were unharmed, and I believe also that such as occupied these houses only during the day might have been comparatively secure.

It is no reply, that the same has not occurred in other streets where ditches have been opened. It *has* occurred, as I think I have it in my power to convince you by an examination of the facts — but if it had not, it would only show that there did not exist in those cases the same catenation of circumstances. The soil thrown up, might have been more clean and destitute of vegetable matter — the heat to which it was exposed might have been less intense — the atmosphere might have been more disturbed by winds, by which the miasms might have been moved off as rapidly as they were evolved. For it will not be forgotten that during these days, from the 24th to the 29th, there was scarcely a breath of air.

Thursday evening there was a slight shower, and during the night a terrific thunder storm, with wind and rain. The wind, which for several days had blown gently from the south, changed now suddenly to a brisk west wind. Since then the temperature of the atmosphere has been much lower.

I have no confidence in the electric origin of cholera, and should not, therefore, under any circumstances, ascribe its accession or departure to electric influences. But here it is certainly a very insufficient explanation of either event. The disease was too limited to have any connection with the general electric state of the atmosphere — and it had already nearly or quite disappeared when the electric explosions occurred.

For myself, I do not doubt, but that the usual concurrence of wind with lightning, has led many to ascribe to the electricity the decrease of the cholera which has occasionally followed; when the *wind* has been in fact the purifying agent to which we were indebted.

In corroboration of the view which I have taken of this matter, I may mention that in 1849, during the summer months, a ditch was commenced in Genesee street, near Michigan, and carried through to Hickory. The ditch was deep and wide, being intended for a sewer. I am informed, by good authority, that as the ditch progressed up the street, the cholera accompanied it, or a little preceded it — and that the cholera continued in this street with extraordinary malignancy until the sewer was finished and the earth removed.

The cholera has definite exciting causes, I affirm, and if we will look we shall sometimes see them. Although it occasionally seeks new localities in its successive annual returns, yet it soon and certainly finds out its old haunts, and there mainly fixes its residence.

In this city we hear of it first, or very soon after it is announced, on the low grounds bordering the canals and the lake; then it creeps along upward and slowly, through the "Hydraulics" to Batavia street, Genesee, and into that portion of the city known as the German quarter — and thence west toward Main street. Finally, as the dry and hot season advances, when its local causes are most intense and abundant, it is found more or less at all points of the city. But nowhere are its victims so numerous, or its attacks so fatal, unless, as in the instance before us, some special local cause is interposed, as in the portions of the town first mentioned.

It prefers Rock street and Evans, because its inhabitants are for the most part poor and debauched, and ripe for any disease; and because its streets are dirty, and its dens reeking with filth. It visits most of the streets lying upon the flats, because the soil being underlaid with clay and not sufficiently drained, produces vegetable miasms, or because the land, by recent excavations and deposits, is so irregularly filled in that a multitude of pools are formed, from which similar emanations arise. It spreads itself tardily into the eastern portion of the town because, although the soil is the same as upon the flats, and although here also much of the water which falls upon it is compelled to disappear by evaporation, yet the land is more elevated and much better drained. It avoids, most of all, the western and northern portions of the town, because, from the peculiar exposure, the ventilation is there most complete. But not only because our prevailing winds are from this direction, does the north-western portion of Buffalo possess a comparative immunity from epidemic diseases, cholera included, but also in part because in such streets as Pearl, Franklin, Delaware, &c.. the soil is sandy and dry, and water does not evaporate even where there are no drains, but rapidly disappears beneath the surface. The same amount of ventilation does not give the inhabitants of Fifth street, nor indeed, I am inclined to believe, of Prospect Hill, the same immunity as is possessed by Pearl, Franklin, and Delaware; and for this reason, that in the places first named the soil will not permit the water to percolate and escape with the same rapidity.

It is probable that the usual concurrence of a clay soil with lime rock, has d to the very common belief that lime water produces cholera. The fac that cholera is found so frequently where lime water is usual, is only another confirmation of my belief that a clay subsoil, by detaining the water upon the surface produces cholera miasms. I wish it to be distinctly understood that I do not regard the clay itself as a source of disease, but rather the water or moist alluvium, or other decomposing matters which lie above it. Where clay is not present, this alluvium alone is sufficient to its production if it is exposed to moisture.

The remote cause of cholera, like the remote cause of the plague, of the yellow fever, &c., has never been ascertained, and I venture to say never will be. It spreads over us like an invisible spirit, whose presence we mysteriously feel, but whose form we can never grasp. It is probable that at the same moment its breath overspreads broad territories, and that its influences are operating no less in the country than in the city. Yet alone and unaided, it is seldom sufficient to produce such results as enable us to recognize its presence. Of what consequence is it to us then, whether we are able to determine the remote cause or not? Of how much less consequence, certainly than that we determine well its near or exciting causes, without whose concurrence the arrows of this pestilence are pointless.

The immediate sources of cholera are before us in this instance, and in a thousand others, and it is our duty as in some sense conservators of the public health, to see and understand them, and by explaining them fully to the people accomplish their removal.

Clean and ventilate the houses; sweep often and thoroughly the streets; fill up the low basins, if possible, with sand; drain well the clay and alluvial soils; remove early in the season all nuisances from the city; and do not leave the cess-pools to be opened and their contents carried through the streets in hot midsummer nights; expose as little as possible during the prevalence of this epidemic, or at the usual seasons of its incursions, the fresh soil to the heats of a fervid sun, especially where alluvium is known to have been buried; let the people live temperately and wisely, and I fully believe this dreadful pestilence would soon cease to visit us, or that at least it would return so shorn of its strength as no longer to baffle the science and skill of our art.

I wish, gentlemen, if you are persuaded that these are truths, that you would do your duty, for this is no trifling matter, and to you the people look for direction. If the causes are visible, so represent to the public officials with whom the sanitary regulations of the city rest, and thus perhaps by your good and timely counsels save many valuable lives.

